# The global battle against SARS-CoV-2 and COVID-19 at the third year

**DOI:** 10.7150/ijbs.76035

**Published:** 2022-07-13

**Authors:** Chu-Xia Deng

**Affiliations:** 1 Editor in Chief, International Journal of Biological Sciences; 2 Chair Professor, Faculty of Health Sciences, University of Macau

Since the outbreak of the Coronavirus Disease-19 (COVID-19) pandemic at the end of 2019, SARS-CoV-2 has spread to over 228 countries and territories, infected at least 561,281,981, and caused more than 6,373,902 deaths as to today (July 12, 2022) 1.

In January 2022, the pandemic reached its highest level with infection rate mounting up to over 3 million per day for nearly one entire month. It gradually dropped to about 500,000 per day in the middle of May, but increased in recent weeks. Meanwhile, the daily deaths declined from over 12,000 cases in January to about 1,500 cases as of today. However, in recent weeks, Omicron BA.4 and Omicron BA.5 gradually become dominant, although currently no evidence suggested that they could cause more severe illnesses than previous variants. Thus, the COVID-19 pandemic is far away from over, even we have gained some experiences in reducing the devastating effects of the disease.

During the past two and half years, the International Journal of Biological Sciences has published 2 special issues focusing on COVID-19 in March 2020 2 and April 2021 3, respectively. In the present special issue, we have organized 17 articles to share authors' understanding of SARS-CoV-2 and COVID-19 from various angles.

Kwok^1^ discussed the roles of the nucleic acid-based, antigen-based, and antibody-based diagnostic tests as the first line of defense in fighting against COVID-19, and the considerations on how to apply these diagnostic tests optimally in the community to cope with the ever-changing pandemic conditions. Xue *et al.* 2 analyzed 48 COVID-19 patients and 17 healthy controls and found that the levels of phosphatidylinositol (PI) and phosphatidylcholine (PC) could distinguish patients from healthy controls, determine the severity of the patients, and thus predict disease aggravation.

Omicron is the most dominant variant of the SARS-CoV-2 virus so far. Analyzing literature systemically, Chen *et al.*^3^ concluded that Omicron-specific humoral immune responses can be enhanced by booster doses of almost all types of vaccines to a certain degree. As all current vaccines are able to elicit robust T cell response against Omicron, they suggested that future focus should be on the development of Omicron variant vaccine, which may induce potent humoral and cellular immune responses simultaneously against all known variants of the SARS-CoV-2 virus. Ye *et al. 4* indicated that the emergence of one after another variant of concern (VOC) poses new challenges for vaccine development. Their work demonstrated that vaccine evasiveness of the emerging VOCs is incomplete and that next-generation VOC-specific vaccines are not only feasible but also required. Li and Luo^5^ also assessed the effectiveness of different COVID-19 vaccines by population studies and neutralization assays and pointed out that as the efficacy of COVID-19 vaccine is expected to decline over time, continued vaccination should be considered to achieve long-term immune protection against coronavirus. As the pandemic developed, asymptomatic infections were increasingly identified through PCR screening of large populations and regular PCR testing of high-risk populations. Cao *et al.*^6^ found that despite lack of overt clinical symptoms, the asymptomatic infections are in active infection with ongoing innate and adaptive immune responses, yet prolonged viral existence. Thus, it is necessary to isolate asymptomatic carriers for management, even though their infectivity may not be strong. Tang *et al.* 7 reported the significant binding between intermediate horseshoe bat ACE2 (bACE2-Ra) and SARS-CoV-2 receptor-binding domain (RBD), thus, the long-term surveillance of intermediate horseshoe bats is vital to minimize spillover risk of SARS-CoV-2.

Suppression of type I interferon (IFN) response is one pathological outcome of the infection of highly pathogenic human coronaviruses. Wu *et al.* 8reported that extracts from coffee leaf are able to block the entrance of SARS-CoV-2 virus to cells more potently than 75% Ethanol. More detailed analysis identified a number of compounds, such as caffeine, chlorogenic acid, quinic acid, and mangiferin, from coffee leaf with an anti-SARS-CoV-2 activity. Following on this line, Wang *et al.*^9^ reviewed tannins, which are polyphenols enriched in wood, bark, roots, leaves, seeds, and fruits of a variety of plants. Because of their properties in antioxidants and anti-SARS-CoV-2, it is believed that tannin derivatives and natural products may be exploited to develop safe and promising strategies in responding to globally emerging new viruses or other pathogens.

He *et al.*^10^ summarized the interplays between the autophagy-lysosome pathway in the host cells and the pathogen SARS-CoV-2 at the molecular level to highlight the prognostic value of autophagy markers in COVID-19 patients and discussed the potential of developing novel therapeutic strategies for COVID-19 by targeting the autophagy-lysosome pathway. Wang *et al.*^11^ reviewed the signaling mechanisms of SARS-CoV-2 Nucleocapsid (N) protein in viral replication, cell death, and inflammation. They concluded that the primary roles of the N protein are to assemble with genomic RNA into the viral RNA-protein (vRNP) complex and to localize to the replication transcription complexes (RTCs) to enhance viral replication and transcription. Yuen *et al.*^12^ reported their work in the identification of ATP citrate lyase (ACLY) as a novel host factor required for efficient replication of SARS-CoV-2 wild-type and variants, including Omicron. Thus, ACLY should be further explored as a novel intervention target for COVID-19.

It has been shown that interfering with nuclear trafficking using pharmacological inhibitors greatly reduces virus infection and virus replication of other coronaviruses was blocked in enucleated cells 4. Chen and Chang^13^ summarized the alternations of nuclear pathways caused by SARS-CoV-2, including the nuclear translocation pathways, the interferon immune response, cytoskeleton regulation, DNA damage response, and nuclear rupture. They also discussed therapeutic strategies that target these pathways with a focus on small molecule drugs that are used in clinical studies.

Many viruses exploit the host lipid metabolism machinery to achieve efficient replication. Studying lipids profile reprogramming *in vitro* and *in vivo* upon SARS-CoV-2 infection, Yan *et al.*^14^ found that phosphatidic acid phosphatase 1 (PAP-1) impairs SARS-CoV-2 replication by affecting the glycerophospholipid metabolism pathway and identified PAP-1 as a potential target for intervention for COVID-19. Metabolic dysfunction-associated fatty liver disease (MAFLD) is a major health issue closely related to metabolic dysfunctions, affecting a quarter of the world's adult population 5. The association of COVID-19 with MAFLD has received increasing attention as MAFLD is a potential risk factor for SARS-CoV-2 infection and severe COVID-19 symptoms 6. Chen and Chen 15 provide an update on the interactions between COVID-19 and MAFLD and its underlying mechanisms. They indicated that SARS-CoV-2 infection can promote the occurrence and development of MAFLD through multiple pathways, including direct cytotoxicity of SARS-CoV-2, hypoxia mediated liver injury, drug-induced liver injury, systemic inflammatory response syndrome (SIRS), and dysregulated lipid metabolism. Indeed, it is known that SARS-CoV-2 infection could also cause many other long-term effects, collectively called “Long COVID”. Koc *et al.* 16 reviewed the general background of Long COVID, and discussed its risk factors, diagnostic indicators and management strategies. Park *et al.* 17 reviewed anosmia, i.e., impairment of smell caused by olfactory dysfunctions, which is a common symptom of the Long COVID. As SARS-CoV-2 may not invade olfactory receptor neurons, how the olfactory functions are affected remains a mystery pending on future investigation.

We hope that the information in this special issue not only facilitates the current war against the SARS-CoV-2 and COVID-19, but will also be useful for preventing the occurrence of similar events, and for building a healthier and safer world in the future.

## Footnotes

1 Kwok HF. The significance of advanced COVID-19 diagnostic testing in pandemic control measures. Int J Biol Sci 2022; 18(12):4610-4617. doi:10.7150/ijbs.72837.

2 Xue M, Zhang T, Cheng ZJ, *et al*. Effect of a Functional Phospholipid Metabolome-Protein Association Pathway on the Mechanism of COVID-19 Disease Progression. Int J Biol Sci 2022; 18(12):4618-4628. doi:10.7150/ijbs.72450

3 Chen Z, Zhang Y, Wang M, *et al*. Humoral and Cellular Immune Responses of COVID-19 vaccines against SARS-Cov-2 Omicron variant: a systemic review. Int J Biol Sci 2022; 18(12):4629-4641. doi:10.7150/ijbs.73583.

4 Ye ZW, Fan Y, Tang K, Ong CP, Luo C, Chung HL, Leong TL, Liang R, Lui WY, Zhou R, Cheng Y, Lu L, Cheung PHH, Chan JFW, Chen Z, Yuen KY, Yuan S, To KKW, Jin DY. Cross-variant protection against SARS-CoV-2 infection in hamsters immunized with monovalent and bivalent inactivated vaccines. Int J Biol Sci 2022; 18(12):4781-4791. doi:10.7150/ijbs.72109

5 Li T, Luo KQ. Recipients of COVID-19 vaccines face challenges of SARS-CoV-2 variants. Int J Biol Sci 2022; 18(12):4642-4647. doi:10.7150/ijbs.72424.

6 Cao S, Zhang Q, Song L, *et al*. Dysregulation of Innate and Adaptive Immune Responses in Asymptomatic SARS-CoV-2 Infection with Delayed Viral Clearance. Int J Biol Sci 2022; 18(12):4648-4657. doi:10.7150/ijbs.72963.

7 Tang L, Zhang D, Han P, *et al*. Structural basis of SARS-CoV-2 and its variants binding to intermediate horseshoe bat ACE2. Int J Biol Sci 2022; 18(12):4658-4668. doi:10.7150/ijbs.73640.

8 Wu CS, Chiang HM, Chen Y, *et al*. Prospects of Coffee Leaf against SARS-CoV-2 Infection. Int J Biol Sci 2022; 18(12):4677-4689.

9 Wang SC, Chou IW, Hung MC. Natural tannins as anti-SARS-CoV-2 compounds. Int J Biol Sci 2022; 18(12):4669-4676. doi:10.7150/ijbs.74676.

10 He W, Gao Y, Zhou J, Shi Y, *et al*. Friend or Foe? Implication of the autophagy-lysosome pathway in SARS-CoV-2 infection and COVID-19. Int J Biol Sci 2022; 18(12):4690-4703. doi:10.7150/ijbs.72544.

11 Wang W, Chen J, Yu X, Lan HY. Signaling mechanisms of SARS-CoV-2 Nucleocapsid protein in viral infection, cell death and inflammation. Int J Biol Sci 2022; 18(12):4704-4713. doi:10.7150/ijbs.72663.

12 Yuen TTT, Chan JFW, Yan B, *et al*. Targeting ACLY efficiently inhibits SARS-CoV-2 replication. Int J Biol Sci 2022; 18(12):4714-4730. doi:10.7150/ijbs.72709.

13 Chen M, Ma Y, Chang W. SARS-CoV-2 and the Nucleus. Int J Biol Sci 2022; 18(12):4731-4743. doi:10.7150/ijbs.72482.

14 Yan B, Yuan S, Cao J, *et al*. Phosphatidic acid phosphatase 1 impairs SARS-CoV-2 replication by affecting the glycerophospholipid metabolism pathway. Int J Biol Sci 2022; 18(12):4744-4755. doi:10.7150/ijbs.73057.

15 Chen H, Chen Q. COVID-19 Pandemic: Insights into Interactions between SARS-CoV-2 Infection and MAFLD. Int J Biol Sci 2022; 18(12):4756-4767. doi:10.7150/ijbs.72461.

16 Koc HC, Xiao J, Liu W, *et al*. Long COVID and its Management. Int J Biol Sci 2022; 18(12):4768-4780. doi:10.7150/ijbs.75056.

17 Park JW, Wang X, Xu RH. Revealing the mystery of persistent smell loss in long COVID patients. Int J Biol Sci 2022; 18(12):4795-4808. doi:10.7150/ijbs.73485.

## References

[1] COVID-19 CORONAVIRUS PANDEMIC.

[2] Deng CX (2020). The global battle against SARS-CoV-2 and COVID-19. Int J Biol Sci.

[3] Deng CX (2021). The continued global battle against SARS-CoV-2 and COVID-19. Int J Biol Sci.

[4] Uddin MdH, Zonder JA, Azmi AS (2020). Exportin 1 inhibition as antiviral therapy. Drug Discov Today.

[5] Younossi Z, Anstee QM, Marietti M, Hardy T, Henry L, Eslam M (2018). Global burden of NAFLD and NASH: trends, predictions, risk factors and prevention. Nat Rev Gastroenterol Hepatol.

[6] Portincasa P, Krawczyk M, Smyk W, Lammert F, Di Ciaula A (2020). COVID-19 and non-alcoholic fatty liver disease: Two intersecting pandemics. Eur J Clin Invest.

